# Impaired Bestrophin Channel Activity in an iPSC-RPE Model of Best Vitelliform Macular Dystrophy (BVMD) from an Early Onset Patient Carrying the P77S Dominant Mutation

**DOI:** 10.3390/ijms23137432

**Published:** 2022-07-04

**Authors:** Arnau Navinés-Ferrer, Sheila Ruiz-Nogales, Rafael Navarro, Esther Pomares

**Affiliations:** 1 Fundació de Recerca de l’Institut de Microcirurgia Ocular, 08035 Barcelona, Spain; arnau.navines@fundacionimo.org (A.N.-F.); sheila.ruiz@fundacionimo.org (S.R.-N.); rafael.navarro@imo.es (R.N.); 2 Departament de Genètica, Institut de Microcirurgia Ocular (IMO), Grupo Miranza, 08035 Barcelona, Spain; 3 Departament de Retina, Institut de Microcirurgia Ocular (IMO), Grupo Miranza, 08035 Barcelona, Spain

**Keywords:** BVMD, BEST1, calcium-activated chloride channel, Ca^2+^-activated Cl^−^ channel, RPE, iPSC-RPE, hPSC-RPE, phagocytosis, bestrophinopathy, Best Disease

## Abstract

Best Vitelliform Macular dystrophy (BVMD) is the most prevalent of the distinctive retinal dystrophies caused by mutations in the *BEST1* gene. This gene, which encodes for a homopentameric calcium-activated ion channel, is crucial for the homeostasis and function of the retinal pigment epithelia (RPE), the cell type responsible for recycling the visual pigments generated by photoreceptor cells. In BVMD patients, mutations in this gene induce functional problems in the RPE cell layer with an accumulation of lipofucsin that evolves into cell death and loss of sight. In this work, we employ iPSC-RPE cells derived from a patient with the p.Pro77Ser dominant mutation to determine the correlation between this variant and the ocular phenotype. To this purpose, gene and protein expression and localization are evaluated in iPSC-RPE cells along with functional assays like phagocytosis and anion channel activity. Our cell model shows no differences in gene expression, protein expression/localization, or phagocytosis capacity, but presents an increased chloride entrance, indicating that the p.Pro77Ser variant might be a gain-of-function mutation. We hypothesize that this variant disturbs the neck region of the BEST1 channel, affecting channel function but maintaining cell homeostasis in the short term. This data shed new light on the different phenotypes of dominant mutations in *BEST1*, and emphasize the importance of understanding its molecular mechanisms. Furthermore, the data widen the knowledge of this pathology and open the door for a better diagnosis and prognosis of the disease.

## 1. Introduction

Bestrophinopathies are a group of inherited retinal dystrophies that typically affect the macular region, impairing central vision. The gene responsible for this group of diseases is *BEST1*. This gene, which comprises 11 exons, is mapped to the 11q13 chromosome and produces a 1758 bp canonical transcript exclusively expressed in the RPE of the adult eye [1,2]. The coding region, starting in the second exon, encodes for the 68kD Bestrophin-1 protein (BEST1), consisting of 585 amino acids with a highly conserved intracellular N-terminal domain containing four transmembrane spanning domains and a long diverse cytosolic C-terminal domain tail [3,4]. BEST1 structures as a homo-pentameric anion channel composed of five BEST1 protomers forming a barrel shaped ion pore. The Ca^2+^ clasps within each protomer form an hydrophobic neck, which is dilated by the binding of cytosolic calcium, allowing the flux of Cl^−^ ions [5,6]. This anion channel is only expressed in the retina by the retinal pigment epithelium (RPE), the cells responsible for maintaining the homeostasis of the photoreceptor cells. Consequently, bestrophinopathies are classed as channelopathies, due to the effects of mutations on the conductance currents through the cell membrane. The lack of BEST1 expression or an incorrect function leads to an early RPE death followed by apoptosis of cones and rods, and the consequent decrease on central high acuity vision [7].

Over 350 different mutations have been identified in the *BEST1* gene, resulting in different clinical manifestations. The most common of these diseases is Best Vitelliform Macular dystrophy (BVMD), a disease with an autosomal dominant pattern of inheritance, a prevalence that ranges from 1:5000 to 1:67,000 and an onset that usually occurs during childhood or early adulthood [7,8,9]. The characteristic presentation of BVMD is by bilateral fundus changes of egg-yolk appearance at the macula in both eyes. The disease, that starts with subtle changes of the retinal pigment epithelium (RPE), appearing on the central retina as a yellowish pigmentation with some granularity defects, evolves to a vitelliform stage with mild vision loss and a decrease on visual acuity (VA) along with other symptoms. Over time, the vitelliform lesion can lead to a vitelliruptive stage, where breakdown of the vitelliform lesion will generate irregular yellow deposits. This deposition, mostly lipofucsin and melanofucsin granulae [10] within the retinal pigment epithelium (RPE), the subretinal space, and the photoreceptor zone can cause a break in the RPE/Bruch’s membrane and a later complication on the choroidal neovascular membrane (CNVM) [11]. Finally, in the atrophic stage, there is a RPE death and loss of photoreceptor cells, leading to widespread geographic atrophy with progressive and irreversible retinal cell loss and the consequent VA decline [7,12,13].

Mutations in *BEST1* have also been associated with other clinically distinct retinal degenerative diseases including Autosomal Recessive Bestrophinopathy (ARB), Autosomal Dominant Vitreoretinochoroidopathy (ADVIRC), Adult Vitelliform Macular Degeneration (AVMD), and Retinitis Pigmentosa 50 (RP50). After BVMD, the most prevalent are ADVIRC and ARB; estimated to have a prevalence of 1:1,000,000 each with an onset range between fourand 40 years old [7]. While AVMD, ADVIRC, and RP50 show an autosomal dominant pattern of inheritance, ARB is, as its name indicates, autosomal recessive; and caused by both bi-allelic homozygous and compound heterozygous variants in *BEST1* [14].

The retinal pigment epithelium is the only cell type in the retina expressing BEST1. It is a monolayer of pigmented cells that lies between the neural retina and the choriocapillaris, directly below the cone and rod photoreceptors. These cells form tight connections with each other and are an essential component of the blood–retinal barrier [15]. The RPE cells are crucial for photoreceptor activity as they are involved in the phagocytosis and degradation of the photoreceptor outer segments (POS) waste and the prevention of the accumulation of photo-oxidative by-products, such as lipofuscin [16]. In addition, RPE cells recycle retinal and other essential substances contained within the POS that are returned to the photoreceptor cells as part of the visual cycle. Changes in cellular pH, Ca^2+^, and ion balance can impact on RPE phagocytosis and affect lysosomal function, therefore, disrupting cell homeostasis and resulting in the accumulation of toxic debris within and around cells, ultimately leading to cell death. The RPE also maintains a healthy retinal environment by secreting signaling molecules, growth factors, neuroprotective factors, and immunosuppressive factors for communication with other tissues [15]. They regulate the buffering of ions in the subretinal space, maintaining ionic balance and pH [17]. To do so, they express several key pumps, transporters, and ion channels at the apical and basal surfaces, such as BEST1.

In RPE, apart from its role as a Ca^2+^ responsive chloride channel, BEST1 may also be involved in other processes such as Ca^2+^ regulation and signaling [18] or eye development [19]. Additionally, the channel is highly permeable to other molecules such as HCO_3_^−^ [20], glutamate [21], and gamma aminobutyric acid (GABA) [22], implying that the channel could potentially serve as a pH sensor/regulator and be involved in neurotransmitter release [7].

Mutations in *BEST1* have been described to affect mRNA expression [23,24], protein expression or folding [25,26], protein localization [27,28,29], or an increased or decreased anion channel function [24,27,30,31]. Each variant leads to a different cellular phenotype and, consequently, to a different clinical manifestation [8]. Based on the results on the molecular mechanisms of several pathogenic *BEST1* variants, Nachtigal et al. [24] proposed a classification of *BEST1* mutations into five different classes (I–V), taking into consideration the effects that each mutation was causing on synthesis, channel function, or protein structure. The mutations where *BEST1* mRNA is degraded (class I), protein is not synthesized (class I) or protein is processed by the proteasome (class II), were associated with an autosomal recessive mode of inheritance. In contrast, when BEST1 was not recognized by the ER quality control and mislocalized (class III) or presented reduced (class IV) or enhanced (class V) anion transport, those mutations were related to a dominant effect on the functionality of the channel and associated with an autosomal dominant inheritance. Phenotypes showing less channel activity have been usually classified as loss-of-function mutations, while those showing more channel activity are deemed as gain-of-function [30,31,32]. The knowledge of each specific mutation has become key for establishing clear genotype–phenotype correlations and achieve a better diagnosis and prognosis of the bestrophinopathies.

To further investigate the impact of *BEST1* mutations in BVMD, we developed a cell line of induced pluripotent stem cells (iPSC) generated from a 35-year old BVMD female with a novel mutation in *BEST1* gene [33]. These iPSCs carry the heterozygous variant c.229C > T (p.Pro77Ser), which has never been described in any other bestrophinopathy patient or neither any of her relatives, as it is a de novo mutation. Through a detailed bioinformatic analysis, it was inferred that the new variant probably had a significant deleterious effect. Moreover, the variant was not present in the public and private databases of human polymorphisms, neither in a cohort of 100 wild type individuals analyzed in our laboratory [33]. For this study, the iPSCs have been differentiated into RPE cells in vitro by small molecule induction [34] and several assays have been carried out in order to evaluate the impact of this mutation.

## 2. Results

### 2.1. Patient’s Case Description

**Figure 1:**
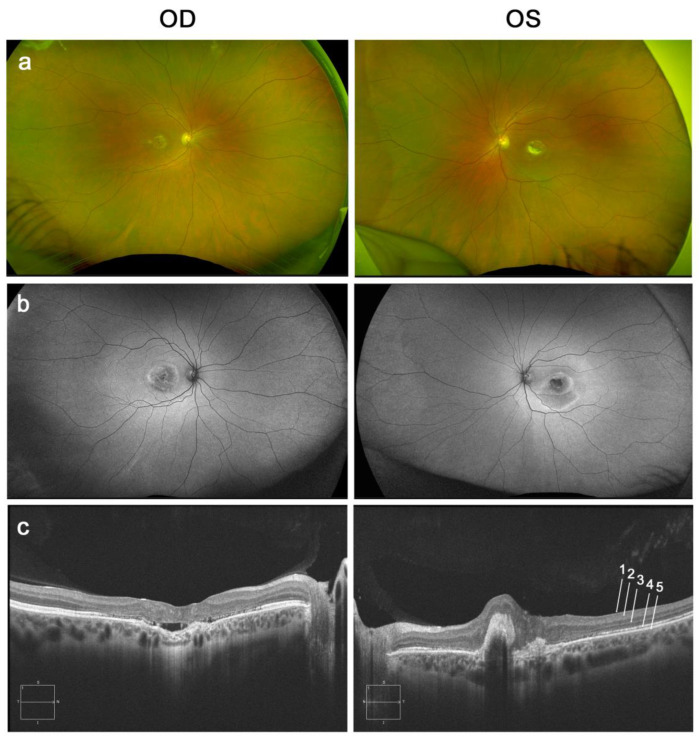
Patient’s clinical description. (**a**) Wide Fundus Retinography and (**b**) wide Fundus Autoflorescence (AF) at the age of 38-years and (**c**) macular Optical Coherence Tomography (OCT) at the age of 35-years of both eyes. Layers of the retina are shown in OCT: 1: Internal limiting membrane, 2: Nerve Fiber Layer, 3: Ganglion cell, inner plexiform, inner nuclear, and outer plexiform layer, 4: Photoreceptor outer nuclear layer, 5: RPE/Bruch’s complex.

Best Disease was diagnosed when the patient was 12-year-old. On the last examination, the patient (38-year-old) presented a Best Corrected Visual Acuity (BCVA) of 20/40 on the right eye (OD) and 20/200 in the left eye (OS). Fundus examination showed retinal pigment epithelium atrophy in the macular area of the OD and a macular scar in the OS due to secondary macular neovascularization (Figure 1). The patient was screened for variants in the *BEST1* gene, and the heterozygous variant c.229C > T (p.Pro77Ser, also referred to in the text as P77S) was detected as the potential cause of the disease.

### 2.2. Fi21/01 iPS-RPE Cells Don’t Show Differential Levels of BEST1 mRNA Expression or Bestrophin Expression

Human induced pluripotent stem cell-derived retinal pigment epithelium (iPSC-RPE) has been used to functionally characterize causal variants of retinal diseases such as BVMD. We previously reported the generation of our patient-derived iPSC [33] and for this work, we used as a control two independent iPS cell lines. Given how much human genetic variation impacts induced pluripotent stem cells, the use of only one control line would be inconsistent for determining significant differences on our patient’s model [35]. Those cell lines were a Wild-Type iPSC line from a patient without any ophthalmologic disease and no genetic variants related to retinal dystrophies (C1) and an iPS cell line derived from a patient with Retinitis Pigmentosa carrying an autosomal dominant *RHO* mutation [36]. As RPE cells do not express rhodopsin, the rod-specific pigment encoded by *RHO* gene [37,38,39,40], we also used this cell line as a control (C2).

**Figure 2:**
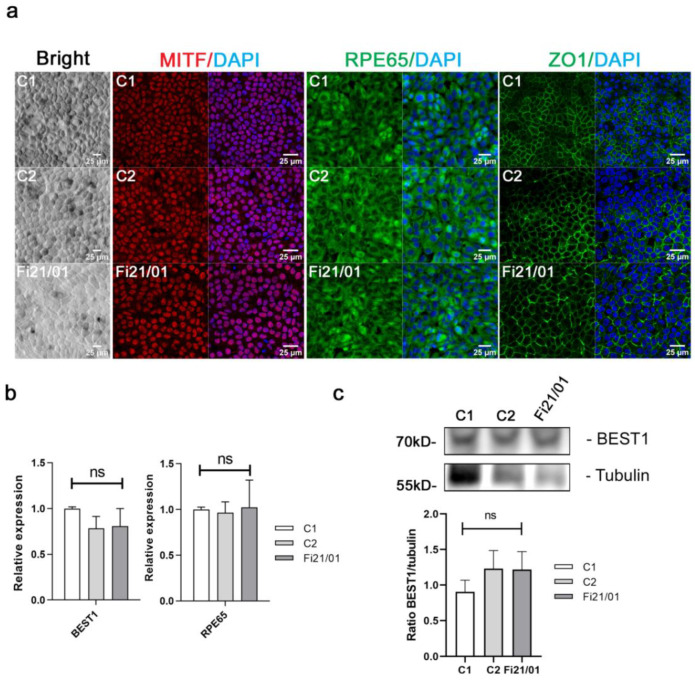
RPE characterization and *BEST1* mRNA and protein expression in iPSC-RPE cells. (**a**) Brightfield images of control 1 (C1), control 2 (C2), and patient’s (Fi21/01) iPSC-RPE cells showing the characteristic cobblestone morphology and expression of MITF (red), RPE65 (green), and ZO-1 (green) on iPSC-RPE cells of both controls and Fi21/01 alone and merged with Hoescht (blue). (**b**) Relative expression of *BEST1* and *RPE65* mRNA from both control iPSC-RPE and Fi21/01 determined by qPCR. (**c**) Protein expression of BEST1 determined by Western Blot from both control iPSC-RPE and Fi21/01 iPSC-RPE. Tubulin was used as a loading control. All experiments were performed at least three times from different biological replicates. Significance was determined by One-way ANOVA (ns = non-significant).

We used a small molecule induction protocol for the differentiation of those iPSC to RPE combining in a sequential manner Nicotinamide, Activin A, and CHIR99021 [34]. After day 56, we obtained an RPE-rich population with the typical dark cobblestone morphology of RPE (Figure 2a, Supplementary Figure S1) and expression of specific RPE markers *MITF*, *RPE65*, and *ZO-1* (Figure 2a, Supplementary Figure S1). There were no clear differences between biological replicates (rounds of differentiation) in the expression or localization of RPE specific markers, although the Fi21/01 cell line consistently showed less pigmentation than control cell lines (Figure 2a).

Expression of *BEST1* mRNA and bestrophin protein expression were determined in our cell line and both C1 and C2 cell lines. We observed no significant differences in the expression of *BEST1* mRNA as determined by qPCR (Figure 2b) and neither in the expression of bestrophin protein as determined by Western Blot (Figure 2c, Supplementary Figure S2). As a control, we checked that all iPSC-RPE cells also expressed the specific RPE marker *RPE65* (Figure 2b).

The Fi21/01 cell line was reanalyzed after RPE differentiation by Sanger sequencing to confirm that the c.229C > T mutation was present (Supplementary Figure S3) and by Whole Exome Sequencing (WES) to rule out any potential retinal-associated genetic variants.

### 2.3. Bestrophin Is Localized Correctly in the Membrane of Mutated iPSC-RPE Cells

**Figure 3:**
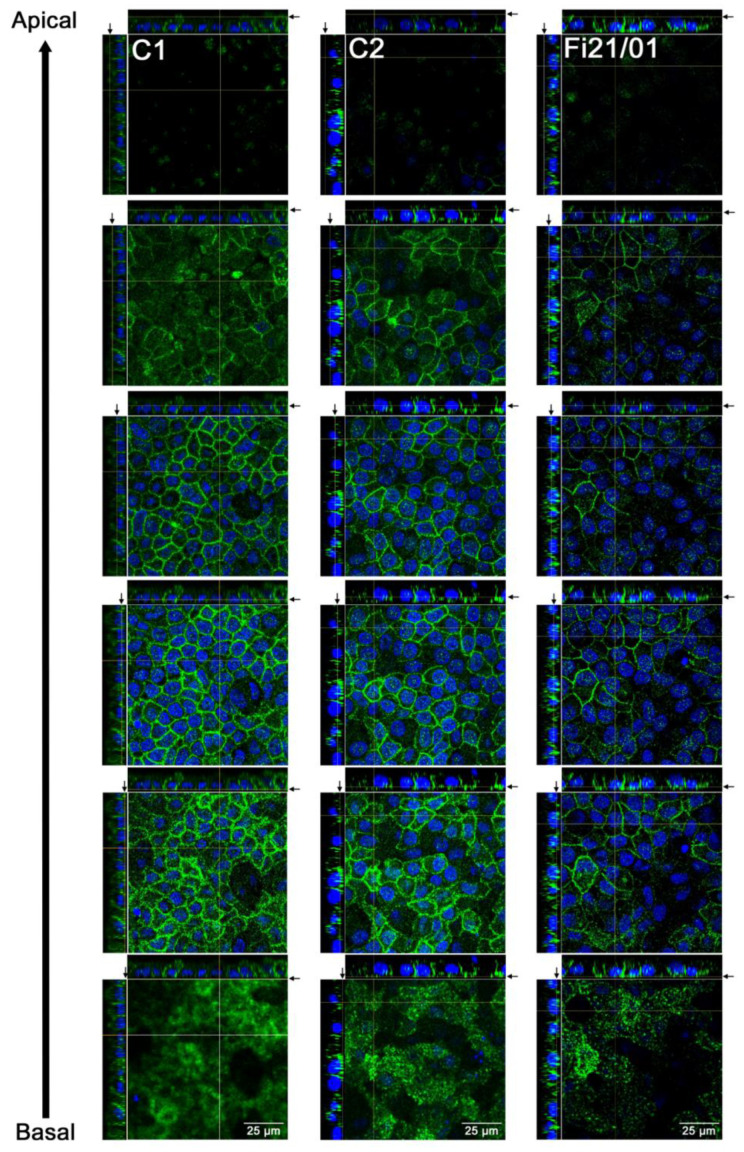
Bestrophin expression from basal to apical regions of iPSC-RPE cells. BEST1 localization determined by immunofluorescence on both controls and Fi21/01 iPSC-RPE and showing of the orthogonal view of the z-stack from different z-stacks from the apical to the basal zone. Arrows in the orthogonal views show the cutting point for horizontal images. Scale bar: 25 µm.

Some mutations in the *BEST1* gene have been reported to induce a mislocalization of the BEST1 channel [28], which is usually found in the basolateral membrane of RPE cells [41]. To determine if the p.Pro77Ser mutation was affecting the localization of BEST1 channel, we investigated its expression by immunocytochemistry in different Z stacks of RPE cell cultures. As shown in Figure 3, the BEST1 expression on the orthogonal view of the Z-stack shows the typical U shape that indicates localization on the basal and lateral membranes of the cell, while showing minimal or no localization in the apical membrane. No differences in protein localization were observed between Fi21/01 and the control lines.

### 2.4. Fi21/01 iPSC-RPE Cells Do Not Show a Significant Apoptotic Profile

**Figure 4:**
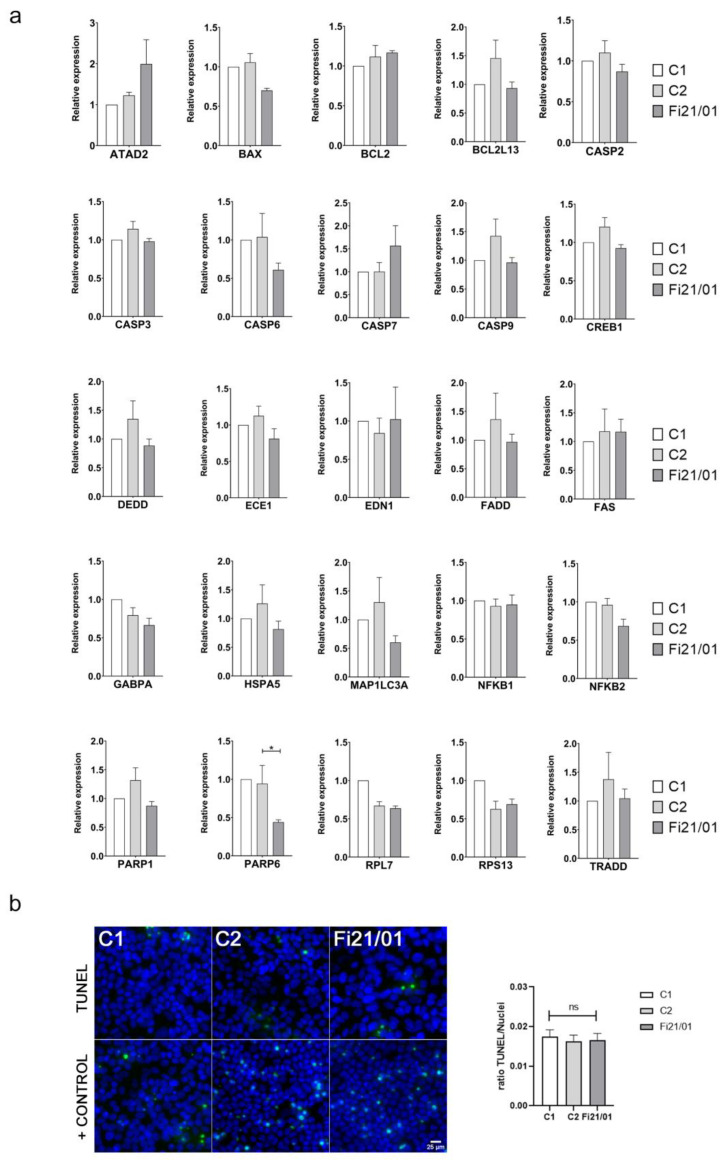
Apoptotic profile on iPSC-RPE cells. (**a**) Relative gene expression of a panel of 25 genes associated with cell apoptosis on both control and Fi21/01 iPSC-RPE. All experiments were performed in triplicates and at least three times with *GAPDH*, *18S*, *HPRT1*, and *GUSB* as housekeeping control genes. (**b**) TUNEL assay of C1, C2, and Fi21/01 iPSC-RPE showing the ratio of TUNEL spots to cell nuclei. All experiments were performed at least three times from different biological replicates. Significance was determined by One-way ANOVA (* = *p* < 0.05, ns = non-significant). Scale bar: 25 µm.

As cell apoptosis is one of the main events underlying the pathology of BVMD, we wanted to determine if our in vitro model of Best Disease would express a different mRNA apoptosis transcriptome than the control cells. For this reason, we checked for an array of 28 genes (Figure 4a) associated with cell apoptosis in human cells or specifically in RPE [42]. Most of the genes did not show statistically significant differences between C1, C2, and Fi21/01 cell lines: *ATAD2* (*p* = 0.1368), *BCL2* (*p* = 0.3737), *BCL2L13* (*p* = 0.1441), *CASP2* (*p* = 0.2846), *CASP3* (*p* = 0.1499), *CASP6* (*p* = 0.2241), *CASP9* (*p* = 0.1569), *DEDD* (*p* = 0.2387), *ECE1*(*p* = 0.1630), *EDN1* (*p* = 0.8744), *FADD* (*p* = 0.5451), *FAS* (*p* = 0.8606), *HSPA5* (*p* = 0.3242), *MAP1LC3A* (*p* = 0.1917), *NFKB1* (*p* = 0.8494), *PARP1* (*p* = 0.0814), *TRADD* (*p* = 0.6072), and *CASP7* (*p* = 0.2788). Some of them showed differences between Fi21/01 and just one control: *BAX* (*p* = 0.0560 for C1 and *p* = 0.0248 for C2), *CREB1* (*p* = 0.7549 for C1 and *p* = 0.0419 for C2), *GABPA* (*p* = 0.0190 for C1 and *p* = 0.4749 for C2), *NFKB2* (*p* = 0.0576 for C1 and *p* = 0.0480 for C2), *RPL7* (*p* = 0.0001 for C1 and *p* = 0.7836 for C2), and *RPS13* (*p* = 0.196 for C1 and *p* = 0.8084 for C2).

Among all those genes, only *PARP6* showed a significant decrease in expression compared to both C1 and C2 (*p* = 0.0224) (Figure 4a). We did not detect any expression of *AHSG*, *AIF1*, or *TNF* in any of our cell lines (data not shown). To determine DNA fragmentation, a distinctive feature of apoptosis, we used a terminal deoxynucleotidyl transferase-dUTP nick end labeling (TUNEL) assay. No differences were found between the control lines and the Fi21/01 line (Figure 4b).

### 2.5. iPSC-RPE Cells with the p.Pro77Ser Mutation Show Increased Halide Entrance

**Figure 5:**
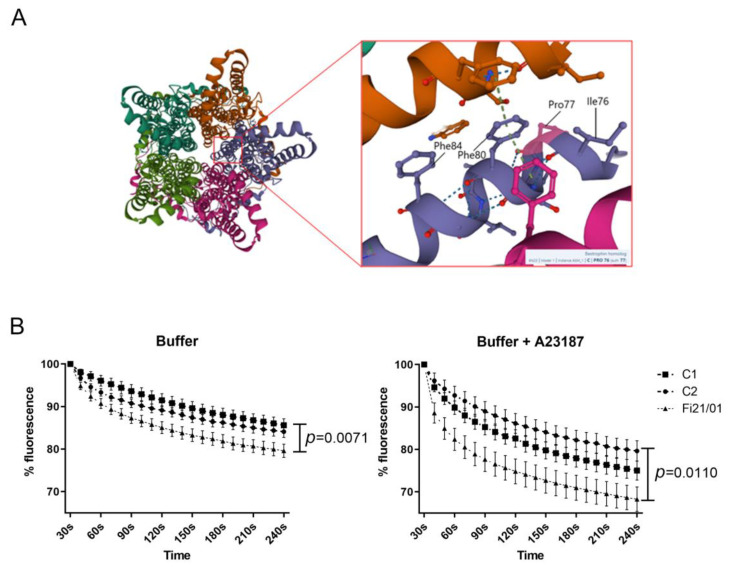
BEST1 structure and halide permeability in iPSC-RPE cells. (**A**) BEST1 channel pentameric structure in a calcium-free closed state (ProteinDB: 6N26) showing the neck residues Phe84, Phe80, and Ile76 critical for neck opening and closure and Pro77, which is mutated in the Fi21/01 patient. (**B**) Percentage of fluorescence from the Premo Halide Sensor shown from 30 s until 240 s (normalized at 100%). Buffer or buffer + A23187 were added at 20 s after the start of the recording. All experiments were performed with 17–21 replicates in three independent experiments with both controls and Fi21/01 iPSC-RPE cells. Significance was determined by Two-way ANOVA with Bonferroni comparison test.

BEST1 protein forms a homopentameric anion channel. The p.Pro77Ser mutation is located in a very conserved region of the protein, the second transmembrane domain that is part of the neck region, which comprises three aminoacids critical for the opening and closure of the channel: Ile76, Phe80, and Phe84 [43] (Figure 5a). The neck serves as both the activation and inactivation gate.

The function of this channel has been usually determined in the literature by measuring the cell membrane conductance through techniques such as patch clamp. In this case, to analyze BEST1 channel function, we transfected the iPSC-RPE cells with the Premo Halide Sensor (Thermofisher Scientific). This method has been used in recent publications with similar non-commercial biosensors [24,27,44]. The sensor, which is introduced in the cells through baculovirus, is based in a yellow fluorescent protein (YFP) sensitive to halide ions such as chloride. Twenty-four hours after transfection of the iPSC-RPE cells, they were stimulated with a buffer containing iodine (I^−^), a substitute anion for chloride, with or without the addition of A23187 (Calcimycin), which promoted the intracellular release of calcium in the cell. When the channel is activated, the iodine ions flow through and they quench the YFP fluorescence in a directly proportional manner. We could observe a quenching of YFP signal, indicating channel activity after the addition of the buffer. There was a significantly higher decrease of the YFP signal in Fi21/01 cells compared to control cells with only buffer addition and also after stimulation with the buffer plus A23187 (Figure 5b). These results pointed to an increased halide entrance in Fi21/01 cells both in a resting state and after calcium release by A23187.

### 2.6. No Differences in the Phagocytosis Capacity of Fi21/01cells Compared to Control

**Figure 6:**
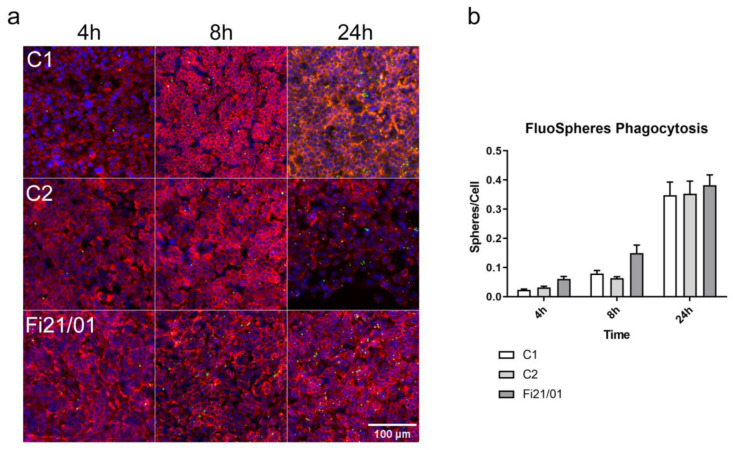
Phagocytosis capacity determination in iPSC-RPE cells. (**a**) Immunofluorescence images of both controls and Fi21/01 iPSC-RPE cells with nuclei staining (blue) and phalloidin-555 (red) and the internalized green-yellow FluoSpheres at different times. (**b**) Quantification of the number of spheres/number of cells at different times (4 h, 8 h, 24 h) of both controls and Fi21/01 iPSC-RPE. Experiments were performed with 8 to 12 replicates on three independent experiments. Significance was determined by Two-way ANOVA with Bonferroni comparison test and no significant differences were observed. Scale bar: 100 µm.

The main function of RPE cells in the retina is to recycle the debris generated during the reception and transduction of the light signal in the photoreceptor outer segments. RPEs engulf an immense amount of material over a life time, disposing of photoreceptor cell waste while retaining useful content [16,45]. To analyze the phagocytosis capacity of our RPE cells, we used yellow-green 0.2 µM amine-coated microspheres, a method that has already been widely used for RPE phagocytosis assays [46,47,48,49,50,51], whichwe incubated in both Fi21/01, C1, and C2 iPSC-RPE cells for 4, 8, and 24 h. A representation of cell distribution by phallodin-alexa555 and microsphere phagocytosis is shown in Figure 6a. We observed that the rate of phagocytosis was increased over time and there were no differences between the patient line and the control lines (Figure 6b).

## 3. Discussion

Among the plethora of inherited macular degenerations, BVMD is the second most prevalent, just after Stargardt’s disease [52]. This autosomal dominant disorder, along with the rest of bestrophinopathies (ARB, ADVIRC, AVMD, and RP50),iscaused by over 350 mutations [53] in *BEST1* with variable expression, different modes of inheritance, and cases of incomplete penetrance [54]. BVMD presents vitelliform lesions that evolve and progress through a number of stages that are different in time and form for each patient, usually leading to vision loss and a decrease in VA. This is not always the case for all patients with a *BEST1* mutation, as some carriers will have normal or near-normal macular findings [9]. The onset of the disease usually occurs during childhood or early adulthood [8] although some cases may even be noted for the first time as late as at 75 years of age [9]. In contrast with BVMD, other dominant mutations in *BEST1* can cause ADVIRC, which initially presents a strongly demarcated 360-degree circumferential hyperpigmented band in the peripheral retina with later changes in the macular region and other complications [55]. ARB, which results from bi-allelic homozygous or compound heterozygous mutations within *BEST1* shows a more aggressive phenotype along with an earlier onset and affectations on the periphery [14]. In this paper, we show the molecular implications behind a non-described mutation (p.Pro77Ser) from a patient with BVMD using human RPE cells obtained from iPSC of the patient.

Among the tools used for studying these diseases, iPSC have arisen recently as one of the most used in vitro human models for retinal dystrophies and also as a promising model for future treatments [56,57]. RPE disorders, specially, appear to be ideal for human iPSC modeling, as this cell type can be easily generated and manipulated.

**Figure 7:**
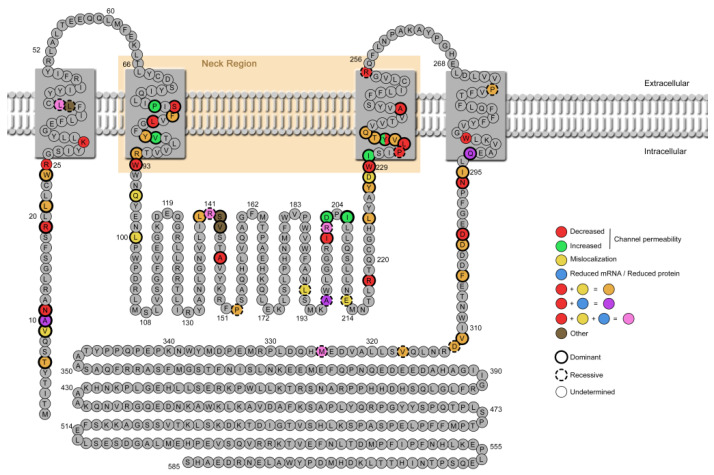
*BEST1*-variants functionally characterized in cellular models. Protein model of bestrophin-1 (based on the structure by Boon 2009 [8]). Functionally characterized mutations from Table 1 are indicated with colors depending on the associated phenotype: decreased or increased anion channel permeability, mislocalization (apical or intracellular), or reduced mRNA or protein (includes misfolding of the protein). Variants reported with more than one effect are also represented. Dominant mutations are represented with a thicker black circle while recessive mutations are represented with a discontinuous circle. The recessive variants tested only in compound heterozygous models are not shown.

The mutation found in our patient is localized in the neck region of BEST1 protein, which forms the activation gate that opens in response to the binding of cytosolic Ca^2+^ ions [43]. Several studies suggest that the neck must widen to allow ion flow and minor movements of the side chain residues lining the neck might be sufficient for ion conduction [6,58]. The walls of the neck present a hydrophobic nature because of the residues I76, F80 and F84, and they act as a hydrophobic seal. This seal may be disturbed by the dramatic change of the 77th proline for a polar aminoacid such as serine. Johnson et al. [28] demonstrated for the first time that the homopentameric structure of BEST1 in patients with dominant mutations was composed by both WT and mutant proteins. In some cases, the expression of an equal ratio of mutant to WT subunits of BEST1 were enough to induce a substantial change in the channel structure and functionality, but in other cases, an allelic imbalance of 4 to 1 was necessary to show a disrupted phenotype. While loss-of-function mutations can behave in a dominant-negative manner, gain-of-function mutations seem to be indeed truly dominant [30]. Then, the allelic imbalance would also be responsible for the incomplete penetrance seen in some healthy carriers with dominant mutations [59]. This realization is very important in sight of the viability of gene augmentation therapies for bestrophinopathy patients. Gene augmentation in both recessive and dominant cases or CRISPR/Cas9-mediated knockdown in the dominant ones, could be a universal treatment strategy for bestrophinopathy patients [30]. Other approaches, such as cellular therapy with healthy iPSC-RPE, or even photoreceptors, open the door for the vision recovery in bestrophinopathy patients or any other cases with genetically associated retinal dystrophies [60]. We observed in our model an increase in anion permeability as shown using a fluorescent YFP sensor for halide ions and stimulation with a iodine-buffer and the calcium ionophore A23187, pointing to that the p.Pro77Ser variant might be a gain-of-function mutation. We were not able to determine the ratio of WT to mutant BEST1 subunits in our iPSC-RPE model, so we are not able to conclude that there is an allelic imbalance present. Most dominant mutations in *BEST1* induce a loss or decrease of the anion channel function (Figure 7, Table 1), while only few of them produce an increase in its activity. It is controversial why some key gain-of-function mutations in BEST1 channel induce a phenotype with peripheral RPE loss, like those seen in ADVIRC or RP, while others induce a macular phenotype. For example, variants V86M, Y236C for ADVIRC, I205T for RP [61], or D203A for BVMD have been shown to be gain-of-function/increased channel permeability [24,31]. Interestingly, it has also been described that W287A mutation showed less channel currents in contrast with the same amino acid mutation W287F, which showed increased channel current [31], highlighting the importance of different aminoacidic changes in the structure and activity of the BEST1 channel.

There are several identified regions that present a high-density for disease-causing mutations (Figure 7): the second transmembrane domain, where the p.Pro77Ser variant resides, which is the bestrophin channel neck region; and the third transmembrane domain that includes the neck neighboring residues [43].These regions are important for channel opening and closure and associated mutations disrupt the pore structure, as already discussed. The C-terminal region next to the fourth transmembrane domain has been found to be critical for channel activation by Ca^2+^. Pathogenic mutations located in this region are probably altering calcium binding [62]. Another key region is the N-terminus, where some mutations have been suggested to disrupt the interaction between N- and C-termini [63]. Finally, mutations in the aperture region of the channel, like V205T, have dramatic effects on ion permeability [64]. Furthermore, while mutations in those critical domains are usually dominant and impair channel function, mutations outside of them are more related to a loss on mRNA or protein expression and a recessive mode of inheritance.

**Table 1:** List of BEST1 aminoacidic changes functionally characterized in cellular models. Each functional characterization describes and references the cellular model in which the experiments were performed, and the phenotype observed (↓ = decreased, ↑ = increased). The associated disease is described and referenced for each aminoacidic change as well as the specific zone where the mutation is found. (pRPE = porcine RPE, fhRPE = fetal human RPE, POS = Photoreceptor Outer Segments, ND = Not Described, Ω = variant described in this manuscript). Asterisks (*) indicate anion conductance determined by YFP chloride sensors instead of patch clamp determination.

AminoacidicChange	MODEL	Localization/Expression	Channel Activity	Others	DISEASE	Domain
**T6P**	pRPE/hiPSC-RPE	Intracellular [65]			BVMD [1]	C-*Terminal*
	MDCKII	Intracellular [28]				
	MDCKII/HEK293	Intracellular [27]	↓* [27]			
**V9M**	MDCKII/fhRPE	Intracellular [66]			BVDM [67]	
**A10T**	HEK293/hiPSC-RPE	↓ protein [32]	↓ [32]		BVMD [67]	
	HEK293/hiPSC-RPE		↓ [30]			
**N11K**	hiPSC-RPE		↓ [24]	lower lysosomal pH (POS, 2w) [24]	BVDM [24]	
**R19C**	HEK293		↓ [63]		BVDM [68]	
**L21V**	MDCKII/HEK293	Intracellular [27]	↓* [27]		BVMD [69]	
**W24C**	MDCKII/HEK293	Intracellular [27]	↓* [27]		BVMD [67]	
**R25C**	HEK293		↓ [63]		ND	
**K30C**	HEK293		↓ [63]		ND	*TM1*
**L40P**	hiPSC-RPE			Decreased fluid flow [70]	ARB [71]	
**L41P**	MDCKII/HEK293	Misfolding [72]	↓ [73]		ARB [73,74]	
	MDCKII	↓ protein/mislocalization [25]	↓ [25]			
**P77S**	hiPSC-RPE		↑* Ω		BVMD [33]	*TM2*
**S79C**	MDCKII/HEK293		↓* [27]		ND	
**F80L**	pRPE/hiPSC-RPE	Intracellular [66]		Reduced Ca^2+^ channel function [66]	BVMD [72]	
	MDCKII/HEK293		↓* [27]			
**L82V**	MDCKII/HEK293		↓* [27]		BVMD [75]	
**Y85H**	MDCKII/HEK293		↓ [73]		BVMD [1]	
	MDCKII	Apical [29]				
	HEK293		↓* [20]			
**V86M**	hiPSC-RPE		↑ [24]		ADVIRC [76]	
**R92C**	HEK293		↓* [20]		BVMD [75]	
**R92S**	MDCKII/HEK293	Intracellular [27]	↓* [27]		BVMD [77]	
**W93C**	HEK293		↓ [78,79]		BVDM [1]	*Loop 2*
	HEK293		↓* [20]			
**Q96R**	MDCKII	Apical [29]			BVMD [80]	
**L100R**	MDCKII	Apical [29]			BVMD [75]	
**L140V**	HEK293	Intracellular [61]	↓ [61]		RP [61]	
**R141H**	MDCKII/HEK293/hiPSC-RPE		↓ [81]		ARB [78,82]	
	HEK293		↓ [78]			
	MDCKII/HEK293	Intracellular [72]				
	MDCKII	Intracellular [28]				
	hiPSC-RPE	↓ protein [24]	↓ [24]			
	MDCKII	↓ protein/mislocalization [25]	↓ [25]			
**R141S**	MDCKII	Intracellular [28]			ARB [83]	
**S142G**	fhRPE			Apoptosis [42]	BVMD [84]	
**V143F**	fhRPE			Apoptosis [42]	BVMD [85]	
**A146K**	hiPSC-RPE			Decreased fluid flow, Impaired phagocytosis (POS, 3, 5 months) [86]	BVDM [86]	
	hiPSC-RPE		↓ [26]			
**A146T**	fhRPE			Apoptosis [42]	ARB [84]	
**P152A**	HEK293		↓ [78]		ARB [78]	
	MDCKII/HEK293	Intracellular [72]				
	MDCKII	Intracellular [28]				
L174Qfs*57	MDCKII	Intracellular [28]			ARB [87]	
**L191P**	MDCKII	Intracellular [28]			ARB [88]	
**A195V**	MDCKII/HEK293	Misfolding [72]	↓ [73]		ARB [72]	
	hiPSC-RPE	↓ protein [26]	↓ [26]			
**R200X**	MDCKII	Intracellular [28]			ARB [78]	
**I201T**	hiPSC-RPE		↓ [89]		BVMD [72]	
**R202W**	MDCKII/HEK293	Intracellular [72]	↓ [73]		ARB [73]	
	MDCKII	↓ protein/mislocalization [25]	↓ [25]			
**D203A**	HEK293		↑ [31]		BVMD [31]	
**I205T**	HEK293		↑ [31]		RP [61]	
	HEK293		↓ [61]			
	HEK293/hiPSC-RPE		↑ [30]			
**E213G**	MDCKII	Intracellular [28]			ARB [28]	
**R218C**	HEK293		↓ [78]		BVDM [67]	
	MDCKII/HEK293		↓* [27]			
	hiPSC-RPE		↓ [24]	lower lysosomal pH (POS, 2w) [24]		
	hiPSC-RPE		↓ [26]			
**R218H**	HEK293/hiPSC-RPE		↓ [32]		BVMD [72]	
	hiPSC-RPE		↓ [44]			
	HEK293/hiPSC-RPE		↓ [30]			
**L224M**	MDCKII/HEK293	Intracellular [27]	↓* [27]		BVMD [77]	
**Y227E**	MDCKII	Apical [29]			BVMD [29]	
	MDCKII	Apical [29]				
	MDCKII/HEK293	Intracellular [27]	↓* [27]			
**D228N**	HEK293	Intracellular [62]			BVMD [61]	
**W229E**	HEK293		↓ [63]		BVMD [63]	
**I230A**	HEK293/hiPSC-RPE		↑ [30]		BVMD [30]	*TM3*
**P233A**	HEK293		↓ [31]		ARB [90]	
**L234P**	HEK293/hiPSC-RPE		↓ [32]		BVMD [44]	
	hiPSC-RPE		↓ [44]			
	HEK293/hiPSC-RPE		↓ [30]			
**L234V**	MDCKII		↓ [91]		BVMD [91]	
**V235A**	hiPSC-RPE	Apical [92]			ADVIRC [93]	
**Y236A**	HEK293		↓ [31]		ADVIRC [55]	
**Y236C**	HEK293/hiPSC-RPE		↑ [30]		ND	
	HEK293		↑ [31]			
**T237R**	MDCKII/HEK293		↓ [73]		BVMD [69]	
	MDCKII/HEK293	Intracellular [27]	↓* [27]			
**Q238R**	hiPSC-RPE	Intracellular [24]	↓ [24]	lower lysosomal pH (POS, 2w) [24]	BVDM [24]	
**A243T**	HEK293/hiPSC-RPE		↓ [30,32]		BVMD [72]	
	MDCKII/HEK293		↓* [27]			
	hiPSC-RPE		↓ [44]			
**A243V**	hiPSC-RPE		↓ [24]	lower lysosomal pH (POS, 2w) [24]	BVMD [69]	
	HEK293		↓ [94]			
**R255Q**	hiPSC-RPE		↓ [91]		ARB [91]	*Loop 3*
**P274R**	hiPSC-RPE	Intracellular [91]	↓ [89]		ARB [85]	*TM4*
	HEK293/hiPSC-RPE		↓ [30]			
**W287A**	HEK293		↓ [31]		ND	
**Q293K**	HEK293/hiPSC-RPE	↓ protein [32]	↓ [32]		BVMD [75]	
	HEK293/hiPSC-RPE		↓ [30]			
**I295del**	hiPSC-RPE	Intracellular [24]	↓ [24]	lower lysosomal pH (POS, 2w) [24]	BVDM [82]	*C-terminal (Ca^2+^ binding)*
**N296H**	hiPSC-RPE		↓ [26]		BVMD [72]	
	hiPSC-RPE			Decreased fluid flow, Impaired phagocytosis (POS, 3, 5 months) [88]	BVDM [72]	
**N296S**	MDCKII		↓ [91]		BVMD [72]	
**D301N**	HEK293		↓ [63]		BVMD [82]	
**D302A**	HEK293/hiPSC-RPE		↓ [30,32]		BVMD [95]	
	hiPSC-RPE	Intracellular [66]				
**F305S**	pRPE/hiPSC-RPE	Intracellular [66]		Reduced Ca^2+^ channel function [66]	BVMD [67]	
	MDCKII/HEK293	Intracellular [27]	↓* [27]			
**V311G**	MDCKII/HEK293	Intracellular [27]	↓* [27]		BVMD [69]	
**D312N**	MDCKII/HEK293	Intracellular [72]	↓ [73]		ARB [73,77]	*C-terminal*
	MDCKII	Intracellular [28]				
	HEK293		↓ [62]			
**V317M**	MDCKII/HEK293	Intracellular [72]	↓ [73]		ARB [78]	
	MDCKII	Intracellular [28]				
**M325T**	MDCKII/HEK293	Intracellular [72]	↓ [73]		ARB [78]	
	MDCKII	Intracellular [28]				
	MDCKII	↓ protein/mislocalization [25]	↓ [25]			
I366fs*18	MDCKII/HEK293/hiPSC-RPE		↑ [81]		ARB [81]	
**L40P + A195V**	hiPSC-RPE			Decreased fluid flow [73]	ARB [71,72]	
**N99K + R141H**	hiPSC-RPE	↓ protein [24]	↓ [24]		ARB [75,82]	
A195V + L197Pfs*26	hiPSC-RPE	↓ mRNA/↓ protein [24]	↓ [24]		ARB [72,96]	
R141H + I366fs*18	hiPSC-RPE	↓ mRNA [23]		Impaired phagocytosis (POS, 5 h) [23]	ARB [77,82]	

There is a normal expression of both *BEST1* mRNA and BEST1 protein in our cell model. This is frequent in missense autosomal dominant mutations, as truncating variants are usually found in a recessive manner in ARB patients. It has been described that these aberrant mutations induce nonsense mediated decay (NMD) of the *BEST1* mRNA [87]. This decrease of mRNA has been observed in several other cases of ARB, with lower mRNA levels and also lower BEST1 protein levels [24,25,26] and it is the reason why carriers of one copy of ARB-related mutations do not develop a *BEST1*-related disease, as they can create a functional channel. In contrast, the ARB patients that are homozygous, or compound heterozygous, carriers lose all channel expression and develop a more aggressive phenotype than BMVD patients. In those cases, the use of *BEST1* gene augmentation could provide such a powerful treatment for ARB patients [32]. Interestingly, Marmostein et al. [23] described that iPSC-RPE derived from ARB patients exhibited impaired POS phagocytosis, displayed similar *BEST1* mRNA levels tocontrol cells but had reduced levels of BEST1 protein. That data suggested that NMD is not always the cause of ARB.

We show that there is a normal BEST1 localization in the basolateral membrane of the Fi2021/01 iPSC-RPE cells in comparison to control cells, and it is neither found in the apical membrane or the intracellular space. Some mutations have shown different mislocalizations of the protein. As seen in Table 1, mutant BEST1 protein can be found, in those cases, in the cytoplasm or the apical domain, for either BVMD or ARB diseases. These mutations prevent the proper delivery of the bestrophin channel to the plasma membrane, thus, impairing channel activity.

Interestingly, some pigmentary changes were observed in our cell model, which havenot been previously described in other iPSC-RPE models of BVMD. There is another case describing less pigmentation in iPSC-RPE cells, which are derived from a patient with RP and carrying mutations in the *USH2A* gene [97]. Although more experiments should be made to investigate this phenotype, it is relevant that hypopigmentation is a sign usually described in bestrophinopathies, retinitis pigmentosa or age-related macular degeneration (AMD) patients [71,97,98,99].

We analyzed the apoptotic profile of our RPE-iPSC cells, which only showed a significant decrease on *PARP6* expression. Some studies have shown that *BEST1* mutations increase *PARP1* and Caspase3 mRNA expression in RPE cells [42]. *PARP1* is the prototypical and founding member of the PARP family, usually activated by DNA damage [100] but *PARP6* has a more complicated role. It has been described that knockdown of *PARP6* promotes cell apoptosis as it is acting as an oncogene in different types of cancers [101,102,103,104]. Overall, and in addition with the TUNEL assay, our results show that there is not a significant increase in apoptosis in Fi21/01 iPSC-RPE in comparison with control cell lines. This can be explained by the fact that a longer exposition to retinal pigments along with a defective function, as it is happening in the patient’s eyes, would be the final cause for the RPE cell death. This phenotype could probably only be seen in vitro by long term feeding of POS [86].

The lack of differences in iPSC-RPE phagocytic competence may also be reflecting the phenotype seen in the patients, as lipofucsin accumulation is only seen after a really long time and can hardly be investigated using an in vitro model in the laboratory. Phagocytosis of amino-coated microspheres is a less specific but an easier method to easily assess RPE phagocytosis, and the spheres can be coated with POS or other molecules for determining a specific function or for drug delivery [51,105,106,107]. Some groups have shown impaired phagocytosis after as little as 5 h in ARB models [23] while in other models of BVMD, the increased accumulation was seen after long term (3.5 months) POS feeding [86]. These differences clearly illustrate the phenotypic disparity observed between those two dystrophies with different onset and severity. Another method for assessing the lack of phagocytic activity would be the determination of lysosomal pH, as shown in other studies [24]. Although we demonstrate that our cell line does not show short-term phagocytosis impairment, longer and more specific experiments would be needed to determine if this cell line shows long-term impairment of phagocytosis.

In conclusion, it is clear that *BEST1* variants identified in patients affected by autosomal dominant bestrophinopathies can lead to different molecular phenotypes depending on both the aminoacidic change and the localization within the BEST1 molecule [7,8]. As variants in a similar region can induce a different disease in the patient, along with different molecular or cellular changes, we cannot conclude that the phenotype caused by a specific variant will be predicted by the region in which this variant is located, but by the specific change that it causes in channel permeability, protein or mRNA expression, and protein localization. It is relevant how many variants have been already described along the same transmembrane domain as our mutation, close to the channel pore (Table 2), but most of them have not been functionally described. In this manuscript, we report for the first time a BVMD variant that induces increased channel permeability in a patient derived iPSC-RPE cell line, and this gives further insight into the phenotype–genotype relationships in this disease.

## 4. Materials and Methods

### 4.1. Generation of Human iPSC

Donor-provided skin fibroblasts were reprogrammed into pluripotent stem cells (iPSC) using the CytoTune^TM^–iPS 2.0 Sendai Reprogramming Kit (Thermofisher Scientific, Waltham, MA, USA—A16517). All iPSCs from this study were characterized by detecting four standard pluripotency markers (SSEA4, Tra-1-60, SOX2 and Nanog) among other specific tests already described [33,36]. iPSC cells were maintained in complete StemFlex Media on Geltrex (Thermofisher Scientific) coated plates. Cells were split on a weekly basis at 1:5–1:10 dilutions using 10 µM Rock Inhibitor (EMD Millipore-Merck Group, Bedford, MA, USA) O/N.

### 4.2. Differentiation of iPSC into RPE

RPE cells were obtained as described in Regent et al. [34] from low passage iPSC. Briefly, cells were incubated in Basal Media (DMEM/F12, 1% Pen/Strep, 1% N2 media supplement, 1% B27 media supplement, 1% non-essential aminoacids (NEAA), and 10% KnockOut Serum Replacement (KSR) with an extra 10% KSR, 50 µM β-Mercaptoethanol, and 10 mM Nicotinamide for days 1–7. Nicotinamide was replaced by 100 ng/mL Activin A for days 8–14 and 3 µM CHIR99021 for days 15 to 42. After that period of time, cells were passed in two-steps using TrypLe (Thermofisher Scientific) for 15 min to remove undifferentiated cells, which have lower adherence to the flask, and up to 45 min to detach the RPE cells. The cells were cultured for two more weeks in RPE media (DMEM/F12, 4% KSR, 50 µM β-Mercaptoethanol, 1% NEAA) and after two passages were checked for specific RPE markers. Sanger sequencing was used to determine that the Fi21/01 cell line was keeping the specific *BEST1* mutation after iPSC-RPE differentiation.

### 4.3. RT-PCR and qPCR

To isolate RNA and synthesize cDNA, iPSC-RPE monolayers were lifted with TrypLe and rinsed in PBS. After counting, the pellet of iPSC-RPE cells was lysed using the SuperScript IV Cells direct Synthesis Kit (Thermofisher Scientific) and RT-PCR was performed following Kit instructions. cDNA yield from iPSC-RPE cells was determined using a Qubit 3.0 fluorometer. All gene expression assays were performed with TaqMan fluorescent probes (Thermofisher Scientific) paired with FAM fluorochrome. Forty cycles of PCR using 5–20 ng of input cDNA were performed on an Applied Biosystems QuantStudio 3 qPCR instrument using TaqMan gene expression master mix (Thermofisher Scientific) in quadruplicates. GAPDH was used as a housekeeping gene.

Gene expressions of apoptosis genes were determined using TaqMan Gene expression Array plates (Thermofisher Scientific) and proceed as described in the manufacturer instructions.

### 4.4. Immunofluorescence

Immunofluorescence was performed in 12-well Geltrex-coated plates or 8-well Ibidi µ-slides (Ibidi GmbH, Planegg/Martinsried, Germany) with confluent iPSC-RPE cells. Cells were rinsed 3 times with PBS and fixed with 4% paraformaldehyde for 15 min, permeabilized with 1% Triton X-100 for 15 min, and blocked with FBS 20% + 0.1% Triton X-100 for 1 h. We used the following antibodies: ZO-1 (1A12) mouse monoclonal antibody (Invitrogen, Waltham, MA, USA), BEST1 (E6-6) mouse monoclonal (Invitrogen), RPE65 (MA1-16578) mouse monoclonal (Invitrogen), and MITF (Ab20663) rabbit polyclonal (Abcam, Cambridge, UK). Primary antibody incubation was performed O/N with 1% FBS at 4 °C. Primary antibodies were tagged with an anti-mouse Alexa-488 secondary antibody (Invitrogen) or an anti-rabbit Alexa-586 secondary antibody (Invitrogen) for 1h at RT. Cell nuclei were stained with DAPI (Thermofisher Scientific) for 10 min.

Images were obtained either with a ZEISS Axio Vert.A1 or a Zeiss LSM980 confocal microscope (Carl Zeiss Sports Optics, Jena, Germany) and processed and quantified by ImageJ software.

### 4.5. In Situ Apoptosis TUNEL Assay

The In Situ Apoptosis TUNEL assay was performed as described by manufacturer’s instructions (Click-iT Plus TUNEL-Alexa Fluor 488, Thermofisher Scientific). As a positive control, fixed cells were incubated for 30 min with 1U of DNase I (Thermofisher Scientific). Five to 10 images were taken from each experiment and the ratio of positive TUNEL spots to cell nuclei was analyzed. Images were obtained with a ZEISS Axio Vert.A1 microscope (Carl Zeiss Sports Optics, Jena, Germany) and processed and quantified by ImageJ software.

### 4.6. Western Blotting

First, 1 × 10^5^ cells were lysed with Pierce RIPA buffer (Thermofisher Scientific) plus Halt Protease Inhibitor (Thermofisher Scientific), incubated at 95 °C with loading buffer and β-Mercaptoethanol, and loaded in Mini-Protean TGX Gels (Bio-Rad Laboratories, CA, USA). Western Blot was performed with the MiniProteanTetraCell System (Bio-Rad Laboratories) following manufacturer instructions and the PVDF membranes were incubated with membrane blocking solution (Life Technologies, Carlsbad, CA, USA) and incubated with anti-BEST1 (PA5-78867) rabbit polyclonal (Invitrogen), and anti-tubulin (11224-1AP) rabbit polyclonal (ProteinTech Group, Rosemont, IL, USA) antibodies for 6 h at RT and with goat-anti-rabbit IgG secondary antibody (Invitrogen) for 2 h at RT.

### 4.7. Phagocytosis Assay

Protocol was adapted from Peng et al., 2017 [123] and Toulis et al., 2020 [46]. Briefly, 2 × 10^5^ cells RPE-iPS cells were seeded in p96 µ-plate wells (IbidiGmbh, Gräfelfing, Germany) and added 5 × 10^6^ FluoSpheres per well (Amine-Modified Microspheres, 0.2 µm, yellow-green fluorescent 505/515, Thermofisher Scientific). Cells were incubated for 4, 8, or 24 h and rinsed with warm PBS six times. Then, they were fixed with 4% paraformaldehyde for 15 min, permeabilized with 1% Triton X-100 for 15 min, and blocked with FBS 20% + 0.1% Triton x-100 for 15 min. Cells were finally stained with alexa Fluor Plus 555 phalloidin (Invitrogen) and DAPI (Thermofisher Scientific). Finally, images were acquired with a ZEISS Axio Vert.A1 (Carl Zeiss Sports Optics) and the cell nuclei and the green spheres were counted with ImageJ.

### 4.8. Anion Channel Activity Determination

To determine the influx of halide ions, we seeded 2 × 10^5^ iPSC-RPE cells in p96 µ-plate wells (IbidiGmbh).The Premo™ Halide Sensor (Thermofisher Scientific), which is based on a yellow fluorescent protein (YFP) molecule sensitive to halide ions, was transduced into the cells through a direct non-cytopathic BacMam delivery as described in the manufacturer instructions. After 16 h, yellow-green fluorescence was checked with a ZEISS Axio Vert.A1 (Carl Zeiss Sports Optics) before starting the experiment. 

For channel stimulation, we added 100 µL of warm 2× Premo Halide Stimulus buffer with or without A23187 (5 µg/mL) (Thermofisher Scientific) to each well of iPSC-RPE in 100 uL of RPE media. Fluorescence was recorded every 1000 ms from 20 s before stimulus addition until 300 s (5 min) and the decrease on fluorescence was determined using ImageJ software.

### 4.9. BEST1 3D Structure

BEST1 3D structure was obtained from Protein Data Bank (ProteinDB: 6N26) deposited by Miller A.N., et al. [43].

### 4.10. Statistics

All experiments were performed at least three times from different biological replicates. Each biological replicate is an iPSC clone differentiated to RPE from a low cell passage. Results were analyzed with GraphPad Prism. All graphs are normalized to C1, show the standard error of the mean (SEM), and statistical significance is determined using One-way or Two-way ANOVA statistical analysis with Bonferroni comparison tests.

## Figures and Tables

**Table 2:** List of *BEST1* variants identified in the transmembrane domain 2 (TM2) along with the aminoacidic change caused by the mutation and the associated bestrophinopathy.

Variant	Aminoacidic Change	Phenotype
c.214T > G	p.Y72D	BVMD [85]
c.217A > C	p.I73L	BVMD [108]
c.217A > T	p.I73F	BVMD [109]
c.218T > A	p.I73N	BVMD [110]
c.219C > G	p.I73M	BVMD [68]
c.223C > T	p.L75F	BVMD [111]
c.224T > C	p.L75P	BVMD [112,113]
c.227T > A	p.I76N	BVMD [85,113]
c.228C > G	p.I76M	BVMD [110]
c.227T > C	p.I76T	BVMD [110]
c.229C > T	p.P77S	BVMD [33]
c.232_233 insT	p.S79FfsX153	BVMD [113]
c.236C > A	p.S79Y	ARB [113]
c.239T > G	p.F80C	BVMD [108]
c.240C > A	p.F80L	BVMD [27,72]
c.238T > G	p.F80V	BVMD [108]
c.241G > A	p.V81M	BVMD [113]
c.241G > T	p.V81L	ARB [113]
c.244C > G	p.L82V	BVMD [27,75]
c.248G > C	p.G83A	BVMD [114]
c.248G > A	p.G83D	ADVIRC [115]
c.250T > G	p.F84V	BVMD [85]
c.253T > C	p.Y85H	BVMD [1,29,116]
c.254A > C	p.Y85S	BVMD [117]
c.256G > A	p.V86M	ADVIRC [55,66,93,118]
c.266T > C	p.V89A	BVMD [76]
c.272C > T	p.T91I	BVMD [72,119]
c.274C > T	p.R92C	BVMD [75,84,120]
c.274C > G	p.R92G	BVMD [121]
c.274C > A	p.R92S	BVMD [27,77]
c.275G > A	p.R92H	BVMD [110,122]

## Supplementary Materials

The following supporting information can be downloaded at: https://www.mdpi.com/article/10.3390/ijms23137432/s1.
