# Supplementary material for: Impaired Bestrophin Channel Activity in an iPSC-RPE Model of Best Vitelliform Macular Dystrophy (BVMD) from an Early Onset Patient Carrying the P77S Dominant Mutation
Source: Int J Mol Sci. 2022 Jul 4;23(13):7432. doi: 10.3390/ijms23137432 (PMC9266689; doi:10.3390/ijms23137432)
Supplement: Supplementary file 1 [file ijms-23-07432-s001.zip › Supplementary Figure S1.pdf]

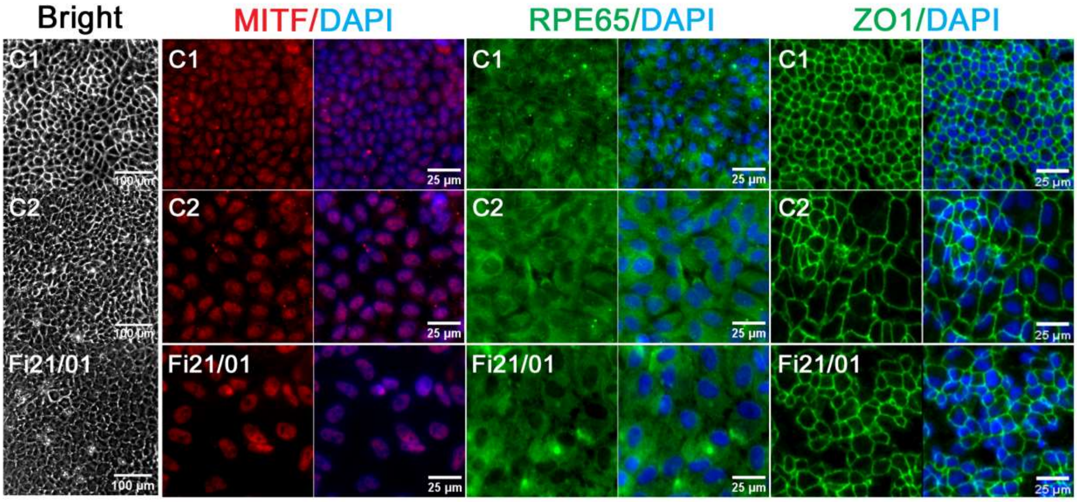

Supplementary Figure S1. Cell morphology (brightfield) and expression of several RPE markers (MITF, RPE65 and ZO-1) in iPSC-RPE cells from both controls and Fi21/01 cells.
