# Supplementary material for: Impaired Bestrophin Channel Activity in an iPSC-RPE Model of Best Vitelliform Macular Dystrophy (BVMD) from an Early Onset Patient Carrying the P77S Dominant Mutation
Source: Int J Mol Sci. 2022 Jul 4;23(13):7432. doi: 10.3390/ijms23137432 (PMC9266689; doi:10.3390/ijms23137432)

Supplementary Figure S2. The data show in Figure 2 is from 2 independent experiments composed of 4 replicates in total. C3 and C4 controls in Blot1 were omitted on the paper because of lack of replicas. In this figure we show the full membranes for BEST1 (70kD) and tubulin (55kD).

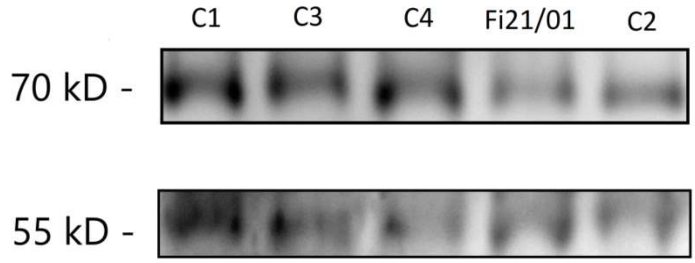

anti-BEST1 (blot1)

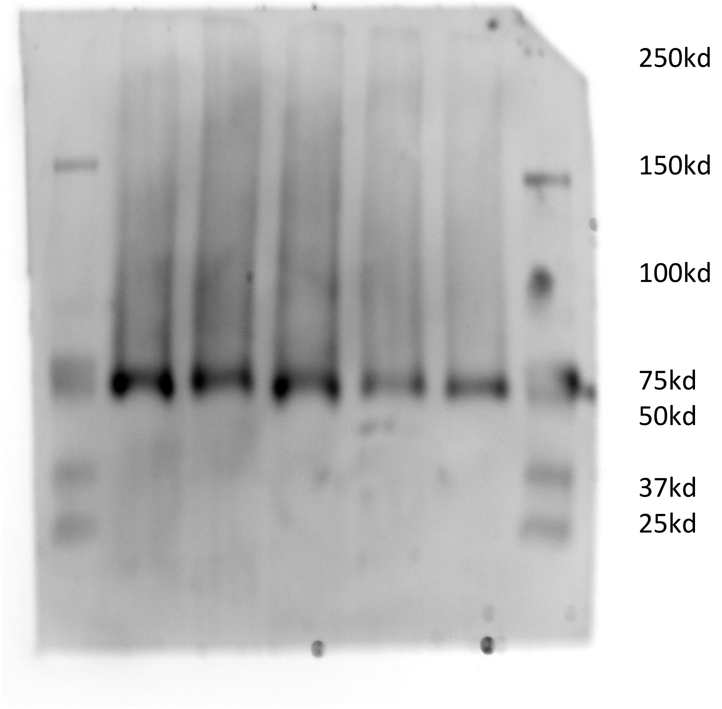

<u>anti-tubulin (blot1)</u>

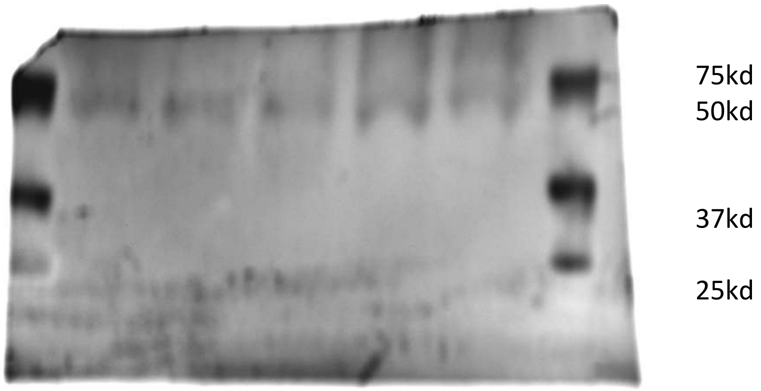

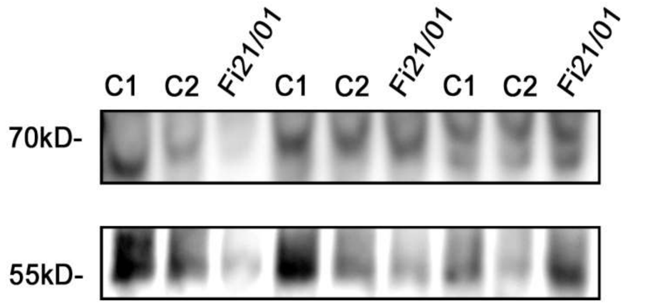

anti-BEST1

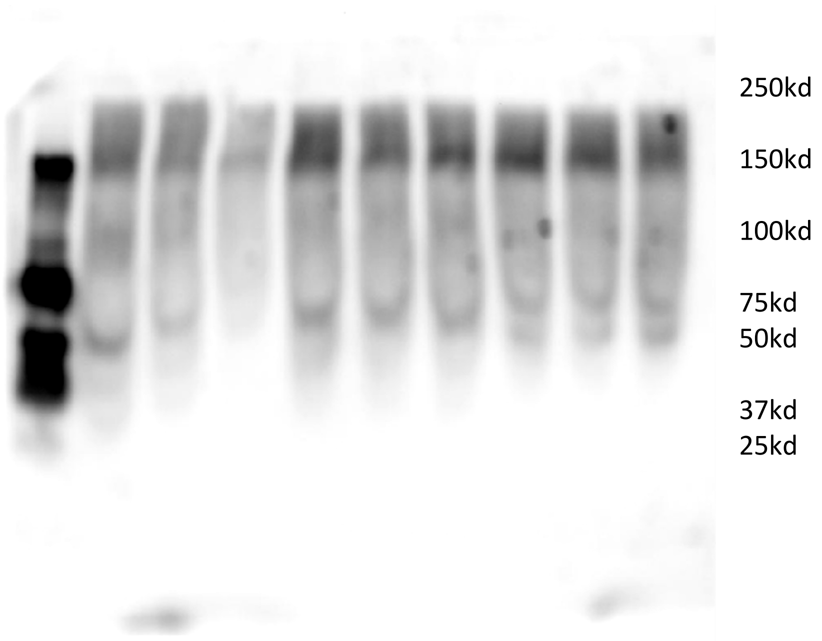

anti-tubulin

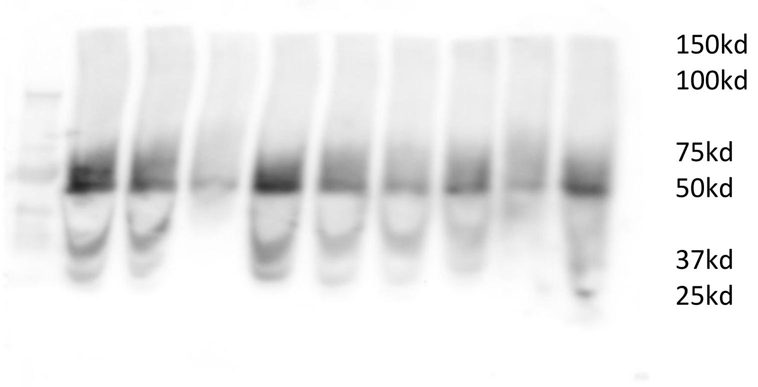

Supplement: Supplementary file 1 [file ijms-23-07432-s001.zip › Supplementary Figure S2.pdf]
