# Supplementary material for: Impaired Bestrophin Channel Activity in an iPSC-RPE Model of Best Vitelliform Macular Dystrophy (BVMD) from an Early Onset Patient Carrying the P77S Dominant Mutation
Source: Int J Mol Sci. 2022 Jul 4;23(13):7432. doi: 10.3390/ijms23137432 (PMC9266689; doi:10.3390/ijms23137432)

Supplementary Figure S3. Forward and reverse Sanger sequences of Fi21/01 iPSC- RPE showing the c.229C>T heterozygous variant in *BEST1*.

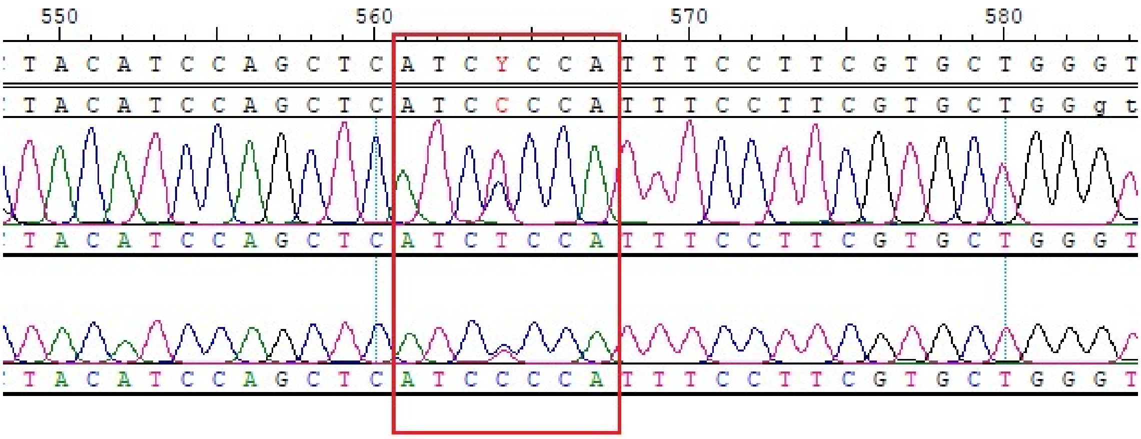

Supplement: Supplementary file 1 [file ijms-23-07432-s001.zip › Supplementary Figure S3.pdf]
